# Osteopontin contributes to virus resistance associated with type I IFN expression, activation of downstream ifn-inducible effector genes, and CCR2^+^CD115^+^CD206^+^ macrophage infiltration following ocular HSV-1 infection of mice

**DOI:** 10.3389/fimmu.2022.1028341

**Published:** 2023-01-04

**Authors:** Adrian Filiberti, Grzegorz B. Gmyrek, Amanda N. Berube, Daniel J. J. Carr

**Affiliations:** ^1^ Department of Ophthalmology, University of Oklahoma Health Sciences Center, Oklahoma City, OK, United States; ^2^ Department of Microbiology and Immunology, University of Oklahoma Health Sciences Center, Oklahoma City, OK, United States

**Keywords:** eye, infection, HSV-1, neovascularization disease, cytokines, macrophage - cell, type - 1 interferons

## Abstract

Ocular pathology is often associated with acute herpes simplex virus (HSV)-1 infection of the cornea in mice. The present study was undertaken to determine the role of early T lymphocyte activation 1 protein or osteopontin (OPN) in corneal inflammation and host resistance to ocular HSV-1 infection. C57BL/6 wild type (WT) and osteopontin deficient (OPN KO) mice infected in the cornea with HSV-1 were evaluated for susceptibility to infection and cornea pathology. OPN KO mice were found to possess significantly more infectious virus in the cornea at day 3 and day 7 post infection compared to infected WT mice. Coupled with these findings, HSV-1-infected OPN KO mouse corneas were found to express less interferon (IFN)-α1, double-stranded RNA-dependent protein kinase, and RNase L compared to infected WT animals early post infection that likely contributed to decreased resistance. Notably, OPN KO mice displayed significantly less corneal opacity and neovascularization compared to WT mice that paralleled a decrease in expression of vascular endothelial growth factor (VEGF) A within 12 hr post infection. The change in corneal pathology of the OPN KO mice aligned with a decrease in total leukocyte infiltration into the cornea and specifically, in neutrophils at day 3 post infection and in macrophage subpopulations including CCR2^+^CD115^+^CD206^+^ and CD115^+^CD183^+^CD206^+^ -expressing cells. The infiltration of CD4^+^ and CD8^+^ T cells into the cornea was unaltered comparing infected WT to OPN KO mice. Likewise, there was no difference in the total number of HSV-1-specific CD4^+^ or CD8^+^ T cells found in the draining lymph node with both sets functionally competent in response to virus antigen comparing WT to OPN KO mice. Collectively, these results demonstrate OPN deficiency directly influences the host innate immune response to ocular HSV-1 infection reducing some aspects of inflammation but at a cost with an increase in local HSV-1 replication.

## 1 Introduction

The normal cornea is a transparent, avascular tissue composed of three layers including the epithelial, stromal, and endothelial layers. Transparency is maintained by the uniformity and size of collagen fibril spacing and diameter, keratocyte crystallin expression, soluble vascular endothelial growth factor (VEGF) receptor 1 and 3, and anti-inflammatory molecules including interleukin (IL)-1 receptor antagonist peptides, pigment epithelial-derived factor and thrombosponin-1 (1) (2–5) (6, 7). Additional mechanisms that regulate the activation of infiltrating lymphocytes and neovascularization include FasL (CD95L) and programmed death ligand-1 (PDL1) which are constitutively expressed by corneal epithelial cells (8, 9). The maintenance of corneal integrity is, in large part, driven by innervation by sensory nerves that express substance P and vasoactive intestinal peptide that also possess immunoregulatory properties (10–12). However, the cornea is not devoid of hematopoietic-derived cells but harbors macrophages, myeloid-derived dendritic cells (DC), plasmacytoid DC (pDC), Langerhans cells and CD34^+^ bone marrow-derived myeloid cells (13–17). These cells tend to be quiescent with low expression of major histocompatibility complex (MHC) class II, and CD40, CD80, and CD86. However, a breach in the “immune-privileged” tissue as a result of trauma or infection can drastically alter the environment and cause irreparable damage.

Herpes simplex virus type 1 (HSV-1) is a highly successful human pathogen that infects the cornea and upon episodes of recurrent reactivation, can lead to a loss of visual acuity and even blindness. In mice, the most common experimental model to study HSV-1 infection, there is a robust inflammatory response to the virus following acute infection. Initially, toll-like receptors (TLR) and innate sensors including IFI-16 expressed by epithelial cells, pDC, and DC perceive the viral nucleic acid from replicating virus in the host cell and elicit the production of pro-inflammatory cytokines, chemokines, and type I IFNs that act directly on virus-infected cells or recruit circulating leukocytes to the site of infection (16, 18–26). The infiltration of innate immune cells into the cornea including neutrophils, macrophages, and natural killer (NK) cells contributes to the resolution of infection but also results in corneal pathology including opacity, denervation, and neovascularization (27–34). In addition to DC and macrophages, corneal epithelial cells initiate expression of MHC class II antigen and may serve as antigen presenting cells (35). Such results extend further as CD4^+^ and CD8^+^ T cells that infiltrate the cornea and facilitate clearance of the insulting pathogen also contribute to tissue pathology (36) (37–39). As a result, the visual axis is significantly compromised which in the case of infected mice, often results in a significant drop in peripheral vision (40).

Early T lymphocyte activation-1 protein (also known as osteopontin, OPN) was originally described as a T cell-derived cytokine that bound macrophages and was associated with severe autoimmunity in mice (41). Using a subjective scoring system to grade herpes stromal keratitis (HSK) severity, one group reported OPN deficient (OPN KO) mice show a significant reduction in the development of HSK coupled with an increase in IL-4 and IL-10 and a loss of IL-12 production by viral antigen-stimulated draining lymph node cell cultures from corneal-infected mice (42). OPN has also been reported to contribute to trauma-induced corneal angiogenesis and wound healing and assist in neutrophil recruitment in fungal-infected mouse corneas likely through secreted isoforms of the parent OPN molecule (43–46). Thus, the immunoregulatory properties of OPN and involvement in cornea pathogenic processes emphasize the role this molecule may play in orchestrating the initial host immune response against ocular HSV-1 infection (47, 48).

Previously, we found a reduction of OPN by 50% using neutralizing antibody led to diminished corneal opacity and damaged collagen in HSV-1-infected mice (49). To further evaluate the role of OPN in the host immune response to infection as well as characterize corneal pathology, we employed OPN KO mice infected with HSV-1. In comparison to wild type (WT) C57BL/6 mice, HSV-1-infected OPN KO mice were found to be more susceptible to infection yet show reduced corneal opacity, neovascularization, and macrophage and neutrophil influx suggesting OPN is a significant contributor to the immune onslaught that results in the degradation of the visual axis.

## 2 Materials and methods

### 2.1 Mice

Male and female C57BL/6J (stock number 000664) and OPN KO (stock number 004936 on a C57BL/6 background) were obtained from The Jackson Laboratory (Bar Harbor, ME., USA) and housed in the Dean McGee Eye Institute vivarium. All animal procedures were approved by the Institutional Animal Use and Care Committee under the protocol, 19-008-AI. Mice were between 7-12 weeks of age at the time of experimental use. Prior to corneal scarification, harvesting tissue, or exsanguinating mice, the animals were deeply anesthetized using ketamine hydrochloride (100 mg/kg, Covetrus North America, Dublin, OH., USA) and xylazine (6.6 mg/kg, Akorn Inc., Lake Forest, IL., USA) administered intraperitoneally. Mice were exsanguinated by cardiac perfusion with 10 ml phosphate buffered saline (PBS, pH = 7.4).

### 2.2 Ocular infection

A stock of HSV-1 (1-10 x 10^8^ plaque forming units, [PFU]/ml, strain McKrae) was propagated in African green monkey kidney (Vero, stock number CCL-81, American Type Culture Collection, Manassas, VA., USA). Anesthetized mice were infected under a dissecting microscope following scarification of the cornea using a 25 ½ gauge needle (Becton Dickinson, Franklin Lakes, NJ., USA) passed over the cornea surface 30x longitudinally and diagonally followed by blotting the surface to remove tear film, and then adding virus (350-500 PFU/cornea) in a 5 µl volume of PBS. Non-infected mice that were scarified served as controls.

### 2.3 Virus plaque assay

Mice were exsanguinated at day 3 or day 7 post infection (PI) and the corneas and trigeminal ganglia (TG) were removed from the animals. The tissue was placed in 2.0 ml microcentrifuge tubes (Midsci, St. Louis, MO., USA) and homogenized using a Tissue tearer (Biospec Products Inc., Mt Pleasant, IL., USA) in 0.5 ml of RPMI-1640 (Gibco Life Technologies, Grand Island, NY., USA) containing 10% fetal bovine serum (FBS, Gibco Life Technologies), antibiotic/antimycotic solution (Thermo Fisher Scientific, Waltham, MA., USA), and gentamicin (Invitrogen, Carlsbad, CA., USA) (referred to as complete media). Viral titers were determined by plaque assay as previously described (50).

### 2.4 Real time reverse transcriptase-polymerase chain reaction

The corneas of WT and OPN KO mice were collected day 3 PI and placed in 1.5-mL GREEN bead lysis tubes (Next Advance, Averill Park, NY., USA) containing 1mL Trizol (ThermoFisher). For RNA isolation, briefly, samples were then homogenized in a Bullet Blender Storm 24 (Next Advance) for 5 min. Samples were incubated 5 min at room temperature to allow complete dissociation of the nucleoproteins complexes, Trizol homogenate was transferred to a new Eppendorf tube and 0.2 mL of chloroform was added. Samples were centrifuged for 15 minutes at 12,000xg at 4°C. The aqueous phase was transferred to in a new 1.5 ml tube. To precipitate the RNA, 0.5 ml of isopropanol was added to the aqueous phase, mixed by inversion three times, and then incubated 10 min on ice followed by centrifugation at 12,000xg for 10 min at 4°C. Following centrifugation, the isopropanol was decanted, and the RNA pellet was resuspended in 1 mL 80% ethanol. The samples were then incubated overnight at 4°C. A final centrifugation was performed at 12,000xg for 5 min at 4°C. The ethanol was decanted, and the tube was air dried at 72°C for 2-3 min. The RNA pellets were resuspended in 25 µL of RNase-free water, and the concentration of RNA was determined using a Nanodrop apparatus (ThermoFisher Scientific). cDNA was generated from the RNA template using an iSCRIPT cDNA synthesis kit according to the manufacturer’s directions (Bio-Rad, Hercules, CA., USA). Forward and reverse oligonucleotide primer sets (**Table 1**) were used to amplify the targeted genes of interest by RT-PCR. A set of proprietary forward and reverse oligonucleotide primers were obtained and validated from a commercial vendor (Bio-Rad) targeting glyceraldehyde 3-phosphate dehydrogenase (GAPDH), IFN-α1, IFN-β, oligoadenylate synthetase (OAS)3, RNA-dependent protein kinase (PKR), RNase L, and tetherin (Bst2) to amplify targeted genes. Melt curves were conducted after all runs to validate a single product. Relative values of gene expression were calculated using the ΔΔCt method following PCR runs on a CFX Connects thermal cycler (Bio-Rad), using GAPDH to normalize samples and CFX Manager software to analyze the data as described (51).

**Table 1:** Oligonucleotide primer pairs for targeted genes of interest.

	Forward	Reverse
IFNa4	5’-TTC TGC AAT GAC CTC CAT CA-3’	5’-GGC ACA GAG GCT GTG TTT CT-3’
mSTING	5’-CCT AGC CTC GCA CGA ACT TG-3’	5’-CGC ACA GCC TTC CAG TAG C-3’
Oas1a	5’-CTT TGA TGT CCT GGG TCA TGT-3’	5’-GCT CCG TGA AGC AGG TAG AG-3’

### 2.5 Protein extraction and suspension array

HSV-1-infected mice were exsanguinated and corneas removed at 12 hr PI or 1, 3, 7, or 15 days PI and placed in 250 µl of PBS containing aproptinin (10 µg/ml, catalog no. A6279; Sigma-Aldrich, Natick, MA., USA), leupeptin (10 µg/ml, catalog no. 1167; Tocris Bioscience, Bristol, UK), and pepstatin (10 µg/ml, Tocris Bioscience) in 1.5-mL GREEN bead lysis tubes (Next Advance, Averill Park, NY., USA). The samples were then homogenized in a Bullet Blender Storm 24 (Next Advance) for 5 min, sonicated in a water bath Bransonic sonicator (Branson Ultrasonics Corp., Danbury, CT., USA) for 5 min, and then homogenized and sonicated again as before. The tubes were then centrifuged (10,000xg, 1 min) in a Micromax RF microcentrifuge (International Equipment Company, Needham Heights, MA., USA). The supernatant was removed from each sample, and the samples were diluted 1:2 in PBS and then frozen back at -80^0^ C until analysis. Total protein content in the clarified supernatant was quantified using a Pierce BCA protein assay kit (catalog no. 23227; Thermo Fisher Scientific). Samples were analyzed for CCL2/MCP-1, CXCL1/KC, IL-6, matrix metalloproteinase (MMP)-2, MMP-8, and VEGF A using customized suspension array kits (Bio-Rad). The limit of detection of each analyte was as follows: CCL2, 10.45 pg; CXCL1, 17.24 pg; IL-6, 1.72 pg; MMP-2, 48.24 pg; MMP-8, 10.63 pg, and VEGF A, 4.55 pg. The Milliplex MAP kits for MMP-2 and MMP-8 were obtained from Millipore (catalog no. MMP3MAG-79K; EMD Millipore, Burlington, MA., USA) whereas kits to detect CCL2, CXCL1, IL-6, MMP-2, MMP-8, and VEGF A were obtained from Bio-Rad. The concentration of each analyte was expressed as pg analyte/mg total protein. OPN was measured using a magnetic Luminex assay kit (catalog no. LXSAMSM-02; R&D Systems, Minneapolis, MN., USA) according to the manufacturer’s instructions.

### 2.6 Flow cytometry

The corneas and submandibular lymph nodes (MLN) were harvested from infected, exsanguinated mice at day 3 or day 7 PI. The MLN were macerated into single-cell suspensions in 1.0 mL complete media (RPMI 1640 + 10% FBS) and kept on ice. Corneas were digested in 0.25 Wümsch units of Liberase TL enzyme (Roche Diagnostics, Indianapolis, IN., USA) suspended in 500 µl complete media for 40 minutes at 37^0^ C with trituration every 10 min. After enzymatic digestion the corneal tissue samples were washed with staining buffer (PBS with 0.5% bovine serum albumin) and along with the MLN samples were then passed through a 40 µm nylon mesh filter (Thermo Fisher Scientific) prior to labeling. Cornea samples were resuspended in 100 µl of staining buffer whereas MLN samples were counted, diluted and finally adjusted to equal number/sample by resuspension in staining buffer prior to labelling. Cell suspensions were first stained with Zombie Aqua for 15 minutes at room temperature and next washed with staining buffer. Subsequently cells were blocked with 1 µl anti-CD16/32 (clone 93; eBioscience, San Diego, CA., USA) for 10 min at 4^0^C and next stained (30 minutes at 4^0^C) with anti-mouse conjugated antibodies (1 µl/antibody/sample) targeting T cell subpopulations (naïve, and effector and central memory T cells) as well as HSV-1 antigen-specific CD8 or CD4 T cells: rat Spark Blue 550-conjugated anti-CD45 (clone 30-F11, Biolegend, San Diego, CA), BV605 conjugated anti-CD3ϵ (clone: 145-2C11) APC-Cy7 conjugated anti-CD4, (clone: GK1.5, Biolegend) FITC-conjugated anti-CD8α (clone 53-6.7, Biolegend), PE-Cy5 conjugated anti-CD44 (clone: IM7, Biolegend), APC conjugated anti-CD62L (clone: MEL-14, Biolegend), BV421 conjugated HSV-1 gD tetramer (IPPNWHIPSIQDA, NIH tetramer core facility, Atlanta, GA) or PE conjugated HSV-1 gB (SSIEFARL, NIH tetramer core facility) tetramer. To determine myeloid cell subpopulations, the MLN and corneal cell suspensions were stained with the following anti-mouse antibodies: APC conjugated anti-CD206 (clone: C068C2, Biolegend), APC-Cy7 conjugated anti-Ly6C (clone: HK1.4, Biolegend), BV480 conjugated anti-CD195 (clone: C34-3448, BD Bioscences), BV605 conjugated anti-MHCII (clone: M5/114.15.2, Biolegend), BV650 conjugated anti-CCR2 (clone: SA203G11, Biolegend), PE conjugated anti-CD11b (clone: M1/70, Biolegend), Spark Blue 550 conjugated anti-CD45 (clone: 30-F11, Biolegend), PE-Cy7 conjugated anti-CD115 (clone: AFS98, Biolegend), Pacific Blue conjugated anti-F4/80 (clone: BM8, Biolegend), PerCP-Cy5.5 conjugated anti-Ly6G (clone: IA8, Biolegend), FITC conjugated anti-CX_3_CR1 (clone: SA011F11, Biolegend) and BV786 conjugated anti-CD183 (clone: CXCR3-173, BD Biosciences).

For evaluation of IFN-γ expressing HSV-1-specific CD4^+^ T cells by ELISPOT, 5x10^6^ MLN cells were stimulated with HSV-1 gD peptide (10 µg/ml) overnight, and the plate was then developed to detect IFN-γ-expressing cells as described (52). For evaluation of IFN-γ, granzyme B, and CD107 expressing HSV-1 antigen-specific CD8^+^ T cells, single cell suspensions of MLN cells (3x10^6^ cells) were cultured in polypropylene tubes in 2.0 ml complete media at 37^0^ C, 5% CO_2_. The cells were stimulated with 100 nM phorbol 12-myristate 13-acetate (PMA) and 1 μM ionomycin (both from MilliporeSigma, Burlington, MA., USA) for 6 hr in the presence of brefeldin A (diluted 1:1000) (GolgiPlug, BD Biosciences) and BV421 conjugated anti-CD107a antibody (clone: 1D4B, Biolegend). After completion of cell stimulation, cells were washed with staining buffer and stained with cell surface anti-mouse antibodies: Spark Blue 550 conjugated CD45, BV605 conjugated CD3ϵ, and PE conjugated CD8 (clone: 53-6.7, eBioscience) for 30 minutes on ice. Next, the cells were washed followed by fixation and permeabilization with eBioscience™ Foxp3/Transcription Factor Staining Buffer Set accordingly to manufacturer guidelines. At the permeabilization step, the cells were intracellularly stained with FITC conjugated anti-granzyme B (clone: QA18A28, Biolegend) and APC conjugated anti-IFN-γ (clone: XMG1.2, Biolegend) for 30 min at room temperature. Cells were then washed in 2.0 ml permeabilization buffer (300xg, 5 min) and resuspended in 200 µl staining buffer for subsequent data acquisition by spectral flow cytometry.

The samples were acquired with either a 3-laser flow cytometer MacsQuant (Miltenyi Biotec) or a 4-laser spectral flow cytometer Aurora (Cytek Biosciences, Fremont, CA USA) containing 16 violet, 14 blue, 10 yellow-green and 8 red channels (4L-16V-14B-10YG-8R). For acquisition using MacsQuant, the compensation was set up using the automatic wizard of the instrument software and if necessary, modified *post hoc*. For acquisition using the Aurora flow cytometer, compensation for spectral unmixing was performed by using single stained reference controls and unmixing wizard integrated in SpectroFlo software (Cytek Biosciences). The general spectra pattern for all fluorochromes used as well as complexity and similarity index for the fluorochrome mix was validated prior labelling based on online resources: Cytek Full Spectrum Viewer (website: https://spectrum.cytekbio.com/). Acquired samples were exported as FCS files and were further analyzed using FlowJo software version 10.7.1 (BD Biosciences, Ashland, OR USA).

### 2.7 Analysis of visual axis

Corneal edema, the blink response, neovascularization, and opacity were evaluated by optical coherence tomography, esthesiometry, confocal microscopy, and absorbance respectively, as previously described (53).

### 2.8 Statistics

Data for each group of experiments was analyzed for significance (p<.05) using Prism 9.0 software (GraphPad, San Diego, CA., USA) using multiple t-test and the Holm-Sidak method. Each experiment was repeated 2-6 times with each group consisting of n=2-4 mice/experiment.

## 3 Results

### 3.1 OPN KO mice are more susceptible in the cornea but not TG following ocular HSV-1 infection

**Figure 1:**
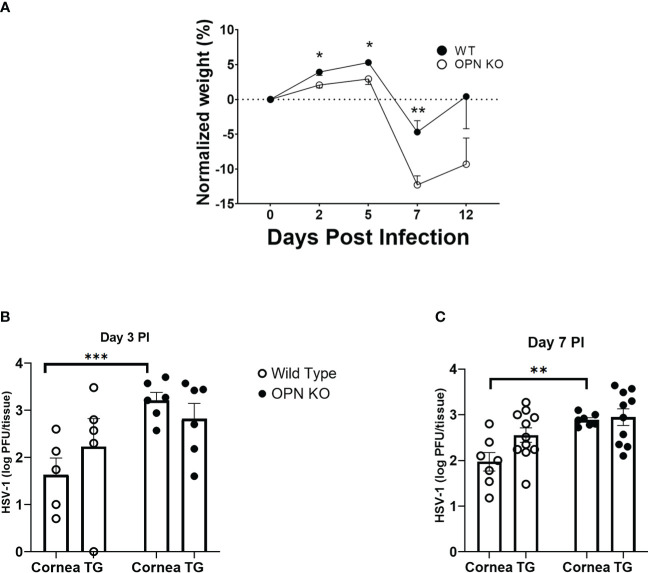
HSV-1 replication is not controlled in the cornea of OPN KO mice. C57BL6 (WT) and OPN KO male and female mice were infected with HSV-1 (350 PFU/cornea). **(A)** At the indicated time PI, the mice (n= 6-24/time point/group) were weighed. The average weight prior to infection (day 0) for WT mice was 22.1 grams and OPN KO mice was 22.4 grams. At day 3 **(B)** or day 7 **(C)** PI, WT and OPN KO mice (n=5-10/group/timepoint) were exsanguinated, and the cornea and TG were removed and processed to determine viral titer by standard plaque assay. *p < .05, **p < .01, and ***p < .001 comparing the two groups at the indicated time point as determined by the Holm-Sidak t-test. Results are depicted as the mean ± SEM.

An early study suggested OPN contributed to the severity of HSV-1 pathology following corneal infection associated with a defective delayed-type hypersensitivity response (42). To determine whether there was an association between ocular disease as reported and local replication of the HSV-1, WT and OPN KO mice were infected and evaluated for weight loss over time and viral titers at day 3 and day 7 PI (**Figure 1**). Initially PI, both WT and OPN KO mice gained weight although there was a significant difference between the two groups of infected mice with WT animals gaining modestly more weight than OPN KO mice up to day 5 PI (**Figure 1A**). However, by day 7 PI OPN KO mice had lost significantly more weight in comparison to the WT counterparts. By day 12 PI, both groups of infected mice began to recover in weight loss from the day 7 PI time point although the OPN KO mice still showed a 10% loss in total weight compared to uninfected (day 0 PI) time point. In terms of virus titer, there was approximately 10-fold more infectious virus recovered in the cornea of OPN KO mice versus WT animals at day 3 (**Figure 1B**) and day 7 (**Figure 1C**) PI. However, there was no difference in the virus load recovered in the TG of infected animals. Moreover, there was no difference in the overall survival rate comparing WT to OPN KO mice with between 15-20% of animals in each group succumbing to infection. Taken together, enhanced replication of HSV-1 in OPN KO mice following ocular HSV-1 infection is restricted to the cornea.

### 3.2 IFNa1 and downstream effector gene expression are suppressed in the cornea of OPN KO mice

**Figure 2:**
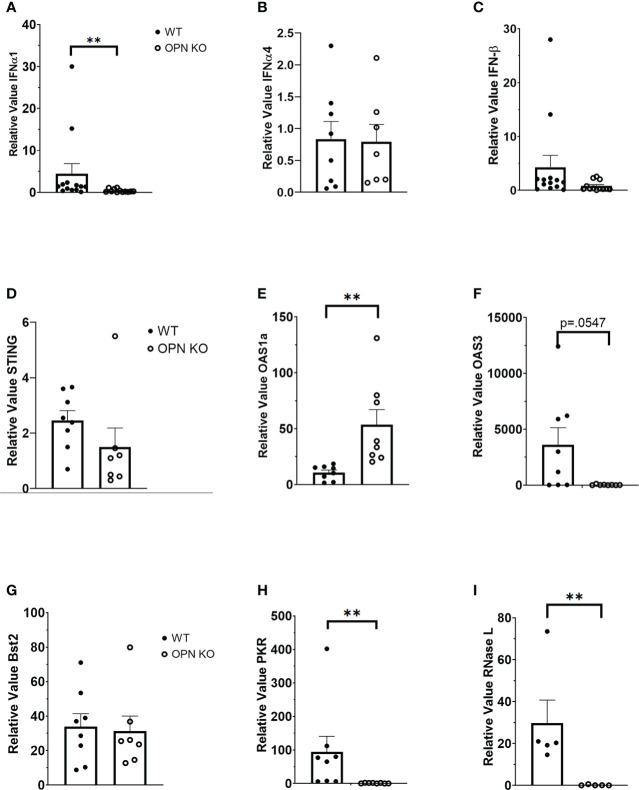
Type I IFN expression is suppressed in the cornea of HSV-1-infected OPN KO mice. WT and OPN KO male and female mice (n=5-13/group) were infected with HSV-1 (350 PFU/cornea). At day 3 PI, the mice were exsanguinated, and the corneas were removed and processed for select gene expression including **(A)** IFN-α1, **(B)** IFN-α4, **(C)** IFN-β, **(D)** STING, **(E)** OAS1a, **(F)** OAS3, **(G)** Bst2 (tetherin), **(H)** PKR and **(I)** RNase L by real time RT-PCR. The results are expressed in relative value compared to uninfected controls (relative value of 1.0) and expressed as the mean ± SEM, **p < .01 comparing the HSV-1 infected WT to OPN KO mice as determined by the Wilcoxon matched-pairs sign rank test.

OPN KO mice have previously been found to be more susceptible to vesicular stomatitis virus (VSV) infection with a reduction in IFN-β expression in the sera of the infected OPN KO mice compared to WT controls (54). Type I IFNs including IFN-β have previously been shown to antagonize ocular HSV-1 infection, and are induced by a number of sensors that can detect and are antagonized by products encoded by the virus (26, 55–57). Since OPN KO mice displayed a loss in virus surveillance in the cornea, we next investigated expression of type I IFN and associated pathways to determine if changes in the levels of expression correlated with HSV-1 susceptibility at day 3 PI. The results show that IFN-α1 (**Figure 2A**) but not IFN-α4 (**Figure 2B**) or IFN-β (**Figure 2C**) were significantly suppressed in the OPN KO mouse corneas compared to WT expression. Investigating downstream pathways activated by type I IFN expression, there was no difference in the relative expression of STING (**Figure 2D**) but oligoadenylate synthetase (OAS)1a was elevated in the cornea of OPN KO mice (**Figure 2E**). Conversely, OAS3a was elevated in the cornea of WT mice in comparison to OPN KO animals although it did not quite reach significance (**Figure 2F**). Previous results reported anti-viral molecules associated with resistance to corneal HSV-1 infection included tetherin (58), OAS/RNase L (59), and PKR (60). Therefore, we investigated the expression of these IFN-driven, downstream effector molecules. In the case of tetherin (*Bst2*), both WT and OPN KO mice expressed similar levels well above the uninfected control animals (relative value = 1) (**Figure 2G**). However, PKR (**Figure 2H**) and RNase L (**Figure 2I**) were significantly reduced in the cornea of OPN KO mice compared to the WT animals. These results strongly correlate the expression of RNase L and PKR to resistance to ocular HSV-1 infection, and suggest OPN influences the expression of both these molecules likely through changes in the activation of IFN-α1.

### 3.3 Myeloid cell infiltration in response to HSV-1 is reduced in the cornea of OPN KO mice

**Figure 3:**
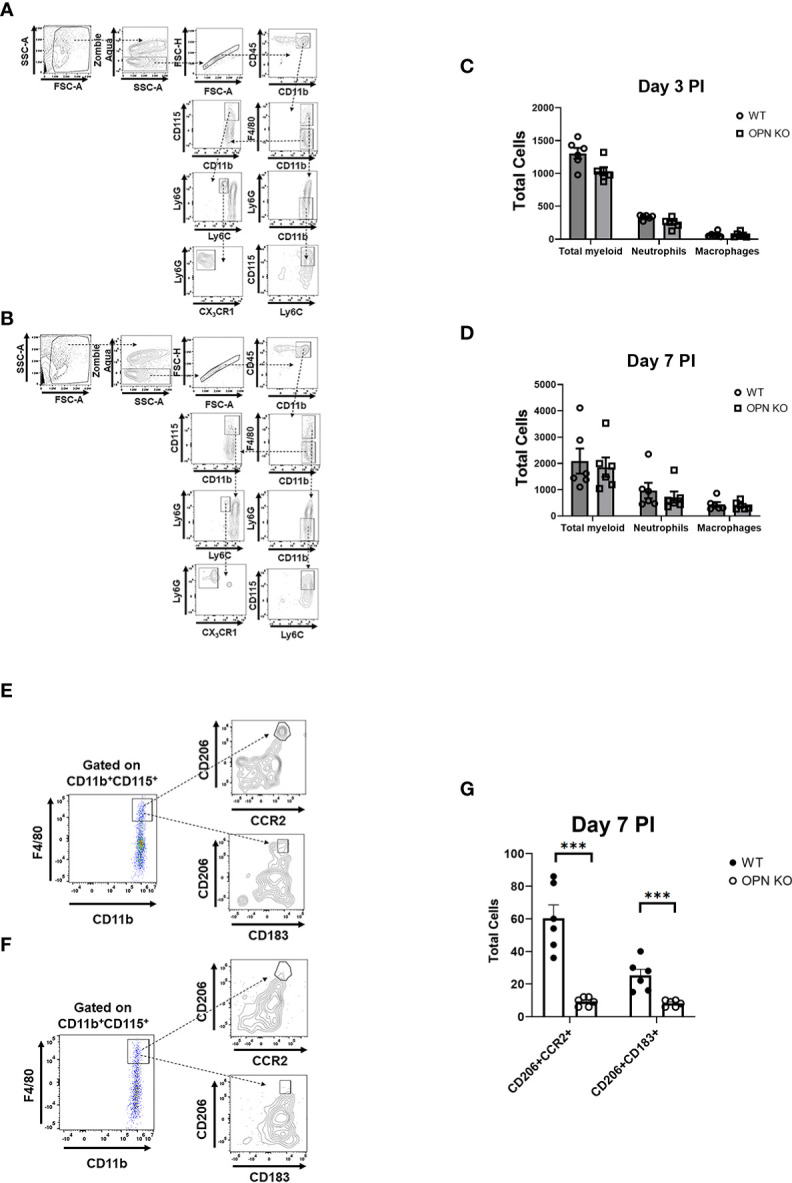
Myeloid-derived cell numbers are reduced in the cornea of OPN KO mice following HSV-1 infection. WT and OPN KO male and female mice (n=6/group) were infected with HSV-1 (350 PFU/cornea). At day 3 or day 7 PI, and corneas were removed from the mice and processed into single cell suspensions in a total volume of 100 µl. The cell suspensions were first stained with Zombie Aqua for 15 minutes at room temperature and next washed with staining buffer. Next, cells were blocked with 1 µl anti-CD16/32 for 10 min at 4^0^C and then stained (30 minutes at 4^0^C) with anti-mouse conjugated antibodies to CD45, CD11b, CD115, CD183, CD206, CX_3_CR1, F4/80, Ly6C, and Ly6G. The samples were then acquired on an Aurora spectral flow cytometer, and the data analyzed using FlowJo software. Gating strategy for total myeloid, neutrophil, and macrophage populations with representative presentations from **(A)** WT and **(B)** OPN KO mice at day 3 PI. Summary of cell counts for total WT and OPN KO myeloid, neutrophil, and macrophage populations for **(C)** day 3 PI and **(D)** day 7 PI. Gating strategy for CD115^+^CD206^+^CCR2^+^CD183^-^F4/80^+^CX_3_CR1^+^ and CD115^+^CD206^+^CCR2^+^CD183^+^F4/80^+^CX_3_CR1^+^ macrophages found in the corneas of **(E)** WT and **(F)** OPN KO mice at day 7 PI. **(G)** Summary of cell counts for the designated macrophage populations from WT and OPN KO mice. Bars represent mean ± SEM ***p<.001. At day 3 PI, p=.06 comparing WT to OPN KO mice as determined by the Holm-Sidak t-test.

Following cornea infection with HSV-1, there is a significant migration of immune cells into the cornea composed initially of myeloid-derived cells, natural killer cells, and γδ T cells of which neutrophils compose the vast majority of the initial myeloid population to enter from circulation (31, 61, 62). Mast cells likely contribute to the recruitment of leukocytes as they are located proximal to the corneal limbal vasculature, are a rich source of chemokines, and rapidly respond to HSV-1 infection through degranulation (63, 64). Moreover, NK cells and macrophages have been reported to play a role in the control of HSV-1 replication in the cornea during acute infection and ensuing HSV keratitis (65–68). Since there was a difference in the amount of infectious virus recovered in the cornea of OPN KO mice compared to WT animals during acute infection, leukocyte infiltration was characterized in the cornea. Initially, myeloid-derived cells were investigated with a focus on neutrophil and macrophage populations. At the early time point post infection, we focused on the total myeloid-derived (CD45^+^CD11b^+^), neutrophil (CD45^+^CD11b^+^F4/80^-^Ly6G^+^Ly6C^lo^CX_3_CR1^-^) and macrophage (CD45+CD11b+F4/80^+^Ly6G^-^Ly6C^+^CD115^+^CX_3_CR1^+^) populating the cornea of WT (**Figure 3A**) and OPN KO (**Figure 3B**) mice. The results show that at day 3 PI there were more total myeloid-derived and neutrophil but not macrophage populations that reside in the cornea of WT mice compared to OPN KO animals although the results did not reach significance (p=.06) (**Figure 3C**). By day 7 PI, all cell populations including the total myeloid-derived, neutrophil, and macrophage populations were nearly equivalent (**Figure 3D**). However, numerous populations of macrophages exist and infiltrate traumatized cornea with two distinct populations associated with corneal wound healing and inflammation (69). In the current study, there was a modest difference in the total number of macrophages residing within the cornea at day 7 PI with more found in the WT compared to OPN KO mouse cornea (**Figure 3D**). Therefore, different populations of macrophages were further analyzed using additional markers including CCR2, CD183, and CD206 along with CD115. Whereas there were no cells detected at day 3 PI using these markers in mouse corneas, by day 7 PI WT (**Figure 3E**) and OPN KO (**Figure 3F**) mouse corneas possessed CD115^+^CD206^+^CCR2^+^CD183^-^F4/80^+^CX_3_CR1^+^ and CD115^+^CD206^+^CCR2^+^CD183^+^F4/80^+^CX_3_CR1^+^ macrophages. Both macrophage phenotypes were found to be significantly reduced in the cornea of OPN KO mice compared to WT animals (**Figure 3G**). Collectively, total myeloid-derived cells, neutrophils, and macrophages were reduced in the cornea of OPN KO mice at day 3 or day 7 PI. The reduction correlates with a reduction in type I IFN expression and inversely, correlates with infectious virus recovered in the cornea at day 3 and day 7 PI in the OPN KO mice.

**Figure 4:**
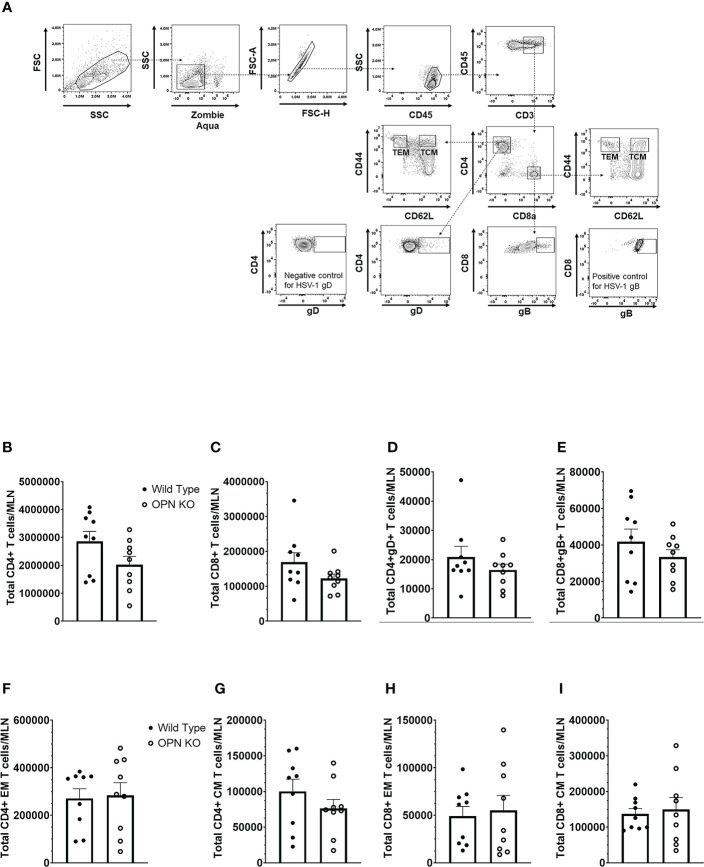
Osteopontin does not influence the development of HSV-1-specific T cells in the draining lymph node during acute infection. HSV-1 infected WT and OPN KO male and female mice (n=9/group) were exsanguinated at day 7 PI, and the draining mandibular lymph nodes (MLN) were removed and processed into single cell suspensions. One million cells were stained with an antibody cocktail including Zombie Aqua to discern viable from dead cells. The gating strategy is shown in panel **(A)** The gD tetramer negative staining control shown in the lower left plot of panel A is: PVSKMRMATPLLMQA conjugated to BV421. The positive control for the gB tetramer staining employed CD8^+^ T cells from gBT-I.1 transgenic mice in which > 95% of the CD8^+^ T cells are specific for gB (74). The total number of CD4^+^ T cells **(B)**, CD8^+^ T cells **(C)**, HSV-1 gD-specific CD4^+^ T cells **(D)**, HSV-1 gB-specific CD8^+^ T cells **(E)**, effector-memory (EM) CD4^+^ T cells **(F)**, central-memory (CM) CD4^+^ T cells **(G)**, EM CD8^+^ T cells **(H)**, and CM CD8^+^ T cells **(I)** per MLN. Bars represent the mean ± SEM summarizing the total of three experiments.

**Figure 5:**
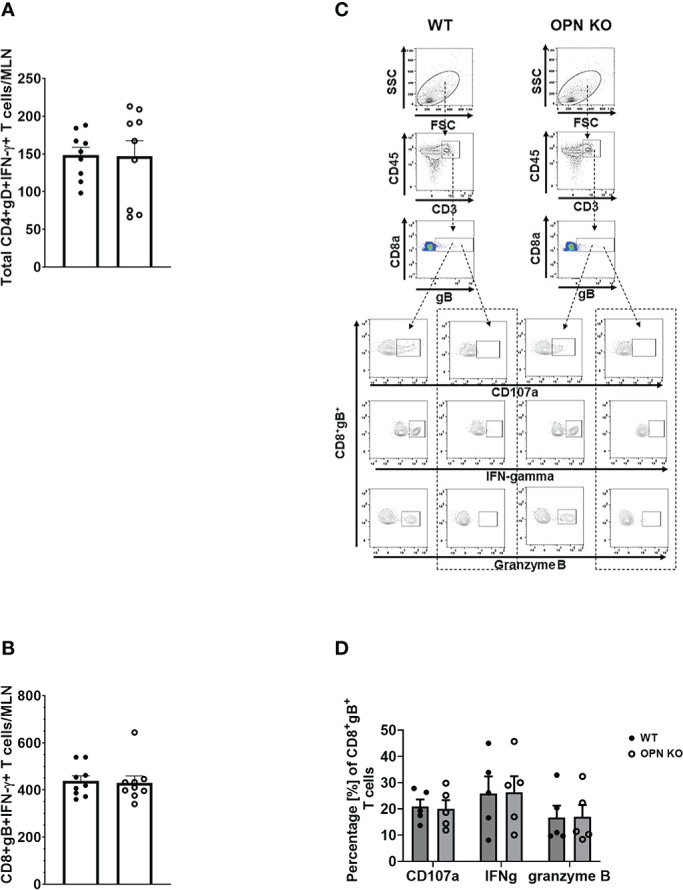
Osteopontin does not alter the function of antigen-specific MLN T cells. HSV-1 infected WT and OPN KO male and female mice (n=5-9/group) were exsanguinated at day 7 PI, and the draining mandibular lymph nodes (MLN) were removed and processed into single cell suspensions. Cells were cultured and stimulated as described under Materials and Methods and during and after stimulation, labeled with an antibody cocktail including Zombie Aqua to discern dead from viable cells. Samples were analyzed by flow cytometry and results processed using FlowJo software. The total number of HSV-1 gD-specific CD4^+^IFN-γ^+^ **(A)** and HSV-1 gB-specific CD8^+^IFN-γ^+^ **(B)** T cells are shown. A representative flow plot assessing CD107a, IFN-γ, and granzyme B expression from stimulated (left panel) and unstimulated (right panel) for each genotype in **(C)** with the summary of the data included in **(D)**. Unstimulated controls possessed fewer than 20 cells. Bars represent the mean ± SEM.

In addition to myeloid-derived cells, T cells are also recruited to the cornea post HSV-1 infection and contribute to tissue pathology and clearance of the virus (38, 70–72). Moreover, OPN is thought to promote a T_H_1 immune response and modulate the development of memory CD8^+^ T cells (42, 73). Therefore, we next investigated the recruitment of T cells to the cornea of infected mice and evaluated the function and development of memory T cells in the draining (mandibular) lymph nodes (MLN) following HSV-1 infection. Unlike myeloid-derived cell populations, CD4^+^ and CD8^+^ T cell recruitment to the cornea was as efficient in OPN KO mice as it was in WT animals. Specifically, the cornea of WT mice harbored 134 ± 30 CD4^+^ T cells compared to 106 ± 28 found in OPN KO corneas at day 7 PI. Likewise, WT mouse corneas contained 96 ± 36 CD8^+^ T cells compared to 60 ± 11 found in OPN KO mouse corneas. Evaluation of the T cell populations within the MLN at the same time point yielded similar results (**Figure 4A**). Specifically, equivalent numbers of total CD4^+^ (**Figure 4B**), total CD8^+^ (**Figure 4C**), HSV-1 glycoprotein (g)D-specific CD4^+^ (**Figure 4D**), HSV-1 gB-specific CD8^+^ (**Figure 4E**), total effector memory CD4^+^ (**Figure 4F**), total central memory CD4^+^ (**Figure 4G**), total effector memory CD8^+^ (**Figure 4H**), and total central memory CD8^+^ (**Figure 4I**) T cells resided in the MLN of WT and OPN KO mice at day 7 PI. To determine if functional changes were found comparing MLN T cells from WT- and OPN KO-infected mice, stimulated MLN T cells were evaluated for IFN-γ expression. The results show that there were no differences in the number of IFN-γ-expressing, HSV-1-specific CD4^+^ (**Figure 5A**) or CD8^+^ (**Figure 5B**) T cells comparing WT to OPN KO mice. Further analysis of the HSV-1 gB-specific CD8^+^ T cell polyfunctional profile (**Figure 5C**) suggested no differences comparing CD8^+^ T cells from the MLN of WT and OPN KO mice (**Figure 5D**) affirmed by SPICE software (**Supplemental Figure 1**). Therefore, we surmise the lack of OPN does not significantly impact the T cell profile in terms of recruitment, number, or function in HSV-1-infected mice.

### 3.4 OPN and IL-6 levels are elevated in the cornea of wild type mice peaking 12-24 hr post infection

**Figure 6:**
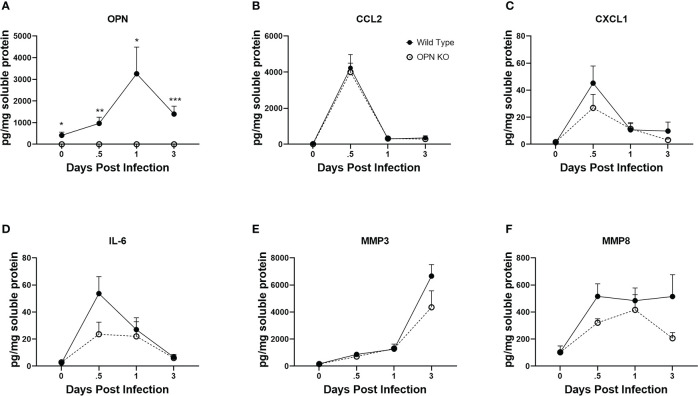
Select cytokine/chemokine levels are not altered in the cornea of OPN KO mice. WT and OPN KO male and female mice (n=5-6/group) were infected with HSV-1 (350 PFU/cornea). At the indicated time point PI, corneas were removed from the mice and processed for analyte expression including **(A)** OPN, **(B)** CCL2, **(C)** CXCL1, **(D)** IL-6, **(E)** MMP3, and **(F)** MMP8 by multiplex suspension array analysis. Each point represents the mean ± SEM ***p<.001, **p<.01, and *p<.05. Uninfected mice served as controls and are represented at the day 0 post infection time point.

Cytokines including chemokines are an important set of molecules in the host immune response to infection as having pro- and anti-inflammatory activity, cell migration promotion and proliferation as well as wound repair. In response to ocular HSV-1 infection, pro-inflammatory cytokines including IL-1, IL-6 and chemokines including CCL2, CCL3, CCL5, CXCL1, CXCL2, CXCL9, and CXCL10 are produced rapidly following infection by resident cells and infiltrating leukocytes (23, 25, 75, 76). OPN has previously been associated with recruitment of neutrophils by upregulation of CXCR2, the receptor for CXCL1 and CXCL2, in a bacterial air-pouch mouse model (77). As myeloid cell infiltration in response to ocular HSV-1 was altered in the absence of OPN, select cytokine and chemokine content was measured at times PI in the cornea of WT and OPN KO mice. As expected OPN was not found in OPN KO mice but was readily detected in uninfected mouse cornea with peak expression detected 24 PI (**Figure 6A**). The chemokines CCL2 (**Figure 6B**) and CXCL1 (**Figure 6C**) and cytokine IL-6 (**Figure 6D**) all peaked earlier at 12 hr PI with no significant differences comparing WT to OPN KO cornea levels although there was a > 2-fold difference between IL-6 with a greater amount detected in the cornea of WT compared to OPN KO corneas at the 12 hr PI time point (p<.09). By comparison matrix metalloproteinase (MMP)3 (**Figure 6E**) and MMP8 (**Figure 6F**) displayed a rise during the early stages of acute infection with no significant differences found comparing WT to OPN KO mice at any time point measured. Therefore, there are no significant differences in select chemokine and cytokine candidates measured that would explain the change in recruitment of myeloid-derived cells into the cornea of OPN KO mice versus WT control animals.

### 3.5 VEGF A levels are elevated early in response to HSV-1

**Figure 7:**
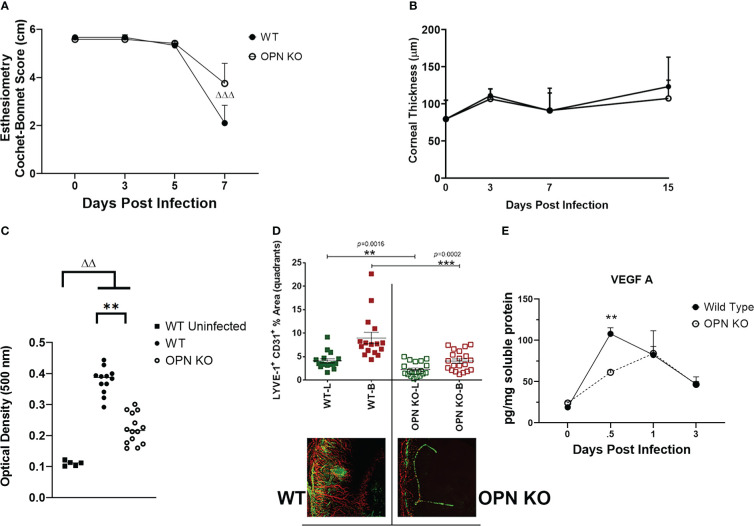
OPN KO mouse corneas display less corneal neovascularization associated with a temporal loss in VEGF A expression. WT and OPN KO male and female mice (n=6-20/group) were infected with HSV-1 (350 PFU/cornea). At the indicated time point PI, the mouse corneas were assessed for **(A)** mechanosensory function by esthesiometry (^ΔΔΔ^p < .001 comparing the day 0 time point to the day 7 post infection time point for the WT mice) and **(B)** corneal thickness by optical coherence tomography. **(C)** At day 7 PI, another group of mice were exsanguinated and the corneas were removed and assessed for opacity. **p < .01, ^ΔΔ^p < .01 comparing the indicated groups as determined by one-way ANOVA and Dunn’s multiple comparison test. **(D)** At day 14 PI, another group of mice were exsanguinated and the corneas were removed, stained for blood (B, red) and lymphatic (L, green) vessels, fixed, and imaged by confocal microscopy. The summary of the area means of images covered by lymphatic and blood vessels ± SEM is shown along with a representative flat mount image of the stained cornea. ***p < .001, **p < .01 comparing the WT to OPN KO blood and lymphatic groups as determined by the Holm-Sidak t-test. **(E)** At the indicated time point PI, corneas were removed from the mice and processed for VEGF A expression by multiplex suspension array analysis. Each point represents the mean ± SEM. Uninfected mice served as controls and are represented at the day 0 post infection time point. **p < .01 comparing the WT to OPN KO mice at 12 hr PI as determined by the Holm-Sidak t-test.

Since there were changes in susceptibility to virus infection and inflammatory response as measured by select myeloid cell infiltration, we next investigated cornea function and pathology comparing WT to OPN KO mice. Initially, the mechanosensory function of the cornea was evaluated during acute infection. Whereas there was no detectable drop in the blink response comparing uninfected mice out to day 5 PI in both groups of infected animals, there was a noticeable drop in the response by day 7 PI with a significant >60% loss in the response in the WT mice (p<.001 comparing day 0 to day 7 PI) and a 30% drop in response in the OPN KO mice (p=0.05316) suggesting both groups had a decline in the blink response but it was more robust in WT animals (**Figure 7A**). Inflammation as a result of leukocyte infiltration and cytokine/chemokine expression often elicits corneal edema and opacity (49, 78). In comparing WT to OPN KO mice, we found no difference in corneal edema measuring thickness by optical coherence tomography at 3, 7, and 15 days PI compared to the uninfected (day 0 PI) time point (**Figure 7B**). However, both WT and OPN KO mice displayed significant corneal opacity at day 7 PI compared to the uninfected WT animals (**Figure 7C**). In addition, infected WT mouse corneas displayed significantly greater opacity compared to infected OPN KO mouse corneas (**Figure 7C**). In mice, acute HSV-1 infection often elicits neovascularization including hem- and lymph-angiogenesis (33, 34). Therefore, corneal neovascularization was evaluated in WT and OPN KO mice at day 14 PI, a time point in which blood and lymphatic vessel development can be readily detected in the normally avascular cornea (79). In the current study, OPN KO mouse corneas displayed a 50% reduction in neovascularization compared to WT animals (**Figure 7D**). The drop in corneal angiogenesis was associated with a temporal loss in VEGF A levels in the cornea of OPN KO mice at 12 hr PI compared to WT mice (**Figure 7E**). These results suggest the absence of OPN leads to a drop in the severity of corneal opacity, neovascularization, and mechanosensory function in response to HSV-1 infection.

## 4 Discussion

OPN is a pleiotropic molecule that has multiple biological effects dictated by OPN processing and the target tissue cell-surface receptors that OPN species binds (80, 81). One such post-translational modification of OPN has been observed to contribute to high affinity binding to histones reducing their cytotoxic activity (82). OPN also directly acts on immune cells contributing to tissue inflammation. For example, macrophage and neutrophil migration is facilitated by expression OPN interaction with integrins (83, 84). In the present study, we found that OPN KO mice infected with HSV-1 showed a muted response in total neutrophil but not macrophage infiltration into the cornea at day 3 PI. Total macrophage numbers were nearly equivalent between the two genotypes at day 3 PI with WT corneas possessing 117 ± 8 total macrophages vs 111 ± 9 total macrophages in OPN KO mouse corneas. By day 7 PI, the number of total macrophages that reside in the WT cornea nearly quadrupled to 446 ± 141 and nearly doubled in the OPN KO mouse corneas at 211 ± 51 total macrophages. Whereas there was no significant difference between the two groups, two subpopulations of macrophages were found to be significantly reduced in the cornea of OPN KO mice; CD115^+^CD206^+^CCR2^+^CD183^-^F4/80^+^CX_3_CR1^+^ and CD115^+^CD206^+^CCR2^+^CD183^+^F4/80^+^ CX_3_CR1^+^ macrophages. In the past and more recently, macrophages that reside or are recruited to the cornea following HSV-1 infection have been broadly defined in terms of the expression of CD11b, F4/80, Ly6C, Ly6G, and/or CD206 or indirectly through genetic manipulation of genes that skew development toward an M1 vs M2 functional phenotype (13, 85, 86). We reasoned further identification of subpopulations of macrophages may identify specific cell types associated with the corneal pathology observed in the WT but not OPN KO mice and not defined by “M1” vs “M2” status as monocytes/macrophages have a high degree of plasticity (87). As CCR2 expressed by monocytes is thought to be a prerequisite to recruitment to inflamed tissue and differentiation of monocytes to macrophages is greatly influenced by the microenvironment (87, 88), additional phenotypic markers would likely facilitate the identification of cell populations associated with pathology or resistance to infection. Of the two major macrophage populations that infiltrated the cornea by day 7 PI (in terms of number), the only distinction in phenotype was whether the cell expressed CD183 (CXCR3). CXCR3-expressing macrophages are reported to possess anti-inflammatory wound healing and antinociceptive attributes or in contrast, a pro-inflammatory signature depending on the tissue and disease (89–93). In the present study, there were more CD183-expressing macrophages in the cornea of HSV-1-infected WT mice aligned with a greater degree of cornea opacity and neovascularization. Previously, we reported a significant reduction in CD115^+^ myeloid-derived cells preserved corneal innervation following HSV-1 infection (94). These cells were also found to be a source of complement component 3 which reportedly contributes to corneal denervation (95). As CD115^+^ cells are a source of OPN and the local reduction in OPN content leads to a loss in HSV-1-induced corneal opacity (49), we reason that a loss in CD115^+^ macrophages contributes to a reduction in corneal opacity in the infected OPN KO mice. Along these lines, macrophages are a source of the neuropeptide substance P (96) that can act through the neurokinin-1 receptor and elicit inflammation and corneal opacity resulting in an inflammatory reflex that can involve the sensory (trigeminal) ganglia (11, 97, 98). Thus, the two major CD115^+^ macrophage populations that infiltrate the cornea during acute HSV-1 infection are likely significant contributors in the corneal opacity that ensues in response to local virus infection and replication.

HSV-1 infection often elicits neovascularization including growth of new blood and lymphatic vessels into the central cornea (34, 99, 100). Macrophages have previously been identified as contributors to vascular proliferation and angiogenesis through the production of pro-angiogenic factors including VEGF-A (101–104). While there is precedence for the physical contribution of macrophages in the genesis of growing corneal vessels as a result of inflammation (105), macrophages do not behave in this manner in response to HSV-1 infection (34). In the present study, there was an direct correlation between the number of CD183^+^ macrophages that have been reported to express VEGF A (93) and neovascularization with fewer CD183^+^ macrophages residing in the OPN mouse cornea. However, another pro-inflammatory factor IL-6 that contributes to HSV-1-induced angiogenesis (100, 106) was not found to be significantly different between WT and OPN KO mice. Whereas there are numerous other factors that play a role in angiogenesis including TNF-α and fibroblast growth factor-2 (79, 107), it is highly likely VEGF-A is the initial stimulus that sets corneal neovascularization into motion in the mouse ocular herpes stromal keratitis model.

A number of cells and factors associated with innate immunity are known to possess potent anti-viral activity including type I IFNs, dendritic cells, natural killer (NK) cells, and macrophages (61, 108). Relative to OPN, a loss of intracellular (i)OPN expression reportedly induces NK cell contraction as a result of an impaired IL-15 response (109). Likewise, iOPN has been found to prevent polyubiquitination of TRAF3, positively regulate IRF3, and enhance IFN-β expression leading to resistance to Sendai virus and vesicular stomatitis virus *in vitro* (54). In the present study, we found OPN KO mice were susceptible to ocular HSV-1 infection with a significant drop in weight during acute infection that correlated with an increase in infectious virus recovered from the cornea but not TG of infected mice compared to WT animals. Such results correlated well with the expression of IFN-α1 which has been found to elicit robust resistance to HSV-1 replication (51). However, STING was not found to be elevated in the cornea of WT compared to OPN KO mice even though previously published data indicate the STING pathway is an integral defense against HSV-1 replication including the cornea and for which the virus attempts to counter through induction of cellular microRNA-24 (20, 58, 110–112). Likewise, the STING-inducible molecule, tetherin (*Bst2*) did not display differential expression in the cornea comparing WT to OPN KO mice. Of considerable note, the OAS system was found to be differentially expressed in the cornea of OPN KO mice in response to HSV-1 infection. OAS1a was found to be significantly elevated whereas OAS3 expression was reduced in the cornea of OPN KO mice post virus infection. Furthermore, the expression of the OAS-dependent effector molecule RNase L was elevated in the cornea of WT mice compared to OPN KO animals suggesting a correlation with OAS3 expression as opposed to OAS1a levels. Such results are consistent with previously published data indicating OAS3 possesses a much higher affinity for double-stranded RNA, and is the principal OAS protein that activates RNase L in response to several RNA and DNA viruses (113). Currently, the location of expression of OAS proteins OAS1a and OAS3 relative to the cell type within the cornea is unknown but will need further assessment to more fully understand the host IFN response to the HSV-1 as OPN is expressed by multiple hematopoietic and non-hematopoietic cell types found in the infected cornea (49).

Lymphatic vessel growth in the avascular cornea is thought to contribute to the regional adaptive immune response to antigen including HSV-1 within the draining lymph node (114, 115). As OPN has previously been found to contribute to the T cell response to HSV-1 (42) and OPN KO mice exhibited a loss in corneal lymphangiogenesis, we investigated the local HSV-1-specific T cell response in the draining (MLN) lymph node during acute infection. However, there was no change in the total number of HSV-1-specific CD4^+^ or CD8^+^ T cells or in the effector or central memory T cells found in the MLN of OPN KO mice in comparison to WT MLN during acute infection. Likewise, the function of T cells in response to HSV-1 antigen was not compromised as determined by IFN-γ expression post stimulation. Therefore, our results suggest the absence of OPN does not alter the T cell response to HSV-1 which is consistent with the results showing no difference in virus titer in the TG comparing WT to OPN KO mice, as T cells play a significant role in the control of virus replication and spread in the TG (116, 117). In conclusion, the present study emphasizes the contribution of OPN in the innate immune response to corneal HSV-1 infection highlighting its absence on a reduction in select type I IFN concentration and activation of downstream pathways. As OPN has biological effects within cells as well as upon secretion and acts upon numerous different cell types (118), the role of OPN within the context of the cornea and HSV-1 infection is indeed complex and will likely require dissecting the biological effects of splice variants residing externally and internally relative to the host cell.

## Data availability statement

The raw data supporting the conclusions of this article will be made available by the authors, without undue reservation.

## Ethics statement

The animal study was reviewed and approved by OUHSC Institutional Animal Care and Use Committee.

## Author contributions

Conceptualization: AF and DC; methodology, AF, GG, AB, and DC; data analysis, AF, GG, AB, and DC; writing original draft, AF, GG, and DC, editing, DC; funding, DC. All authors contributed to the article and approved the submitted version.

## Supplementary material

Supplementary Figure 1 Wild type and OPN KO mice exhibit equivalent polyfunctional CD8^+^ T cell response during acute ocular HSV-1 infection. Data generated from was used to assess for polyfunctionality of CD8^+^ T cells using SPICE software with the results presented as pie charts in panel **(A)**. The average percentage of HSV-1 gB-specific CD8^+^ T cells expressing CD107a, IFN-γ, and granzyme B from WT and OPN KO are displayed. The inner segment segments display cells expressing different combinations of proteins whereas the outer color-coded arcs around each circle show the frequency of cells expressing each protein (CD107a, IFN-γ, and granzyme **(B)**. The polyfunctionality of the cells is indicated in panel B with the data displayed as mean ± SEM.
