# Supplementary material for: Osteopontin contributes to virus resistance associated with type I IFN expression, activation of downstream ifn-inducible effector genes, and CCR2+CD115+CD206+ macrophage infiltration following ocular HSV-1 infection of mice
Source: Front Immunol. 2023 Jan 4;13:1028341. doi: 10.3389/fimmu.2022.1028341 (PMC9846535; doi:10.3389/fimmu.2022.1028341)
Supplement: Supplementary Figure 1 — Wild type and OPN KO mice exhibit equivalent polyfunctional CD8+ T cell response during acute ocular HSV-1 infection. Data generated from was used to assess for polyfunctionality of CD8+ T cells using SPICE software with the results presented as pie charts in panel (A). The average percentage of HSV-1 gB-specific CD8+ T cells expressing CD107a, IFN-γ, and granzyme B from WT and OPN KO are displayed. The inner segment segments display cells expressing different combinations of proteins whereas the outer color-coded arcs around each circle show the frequency of cells expressing each protein (CD107a, IFN-γ, and granzyme (B). The polyfunctionality of the cells is indicated in panel B with the data displayed as mean ± SEM. [file Image_1.pdf]

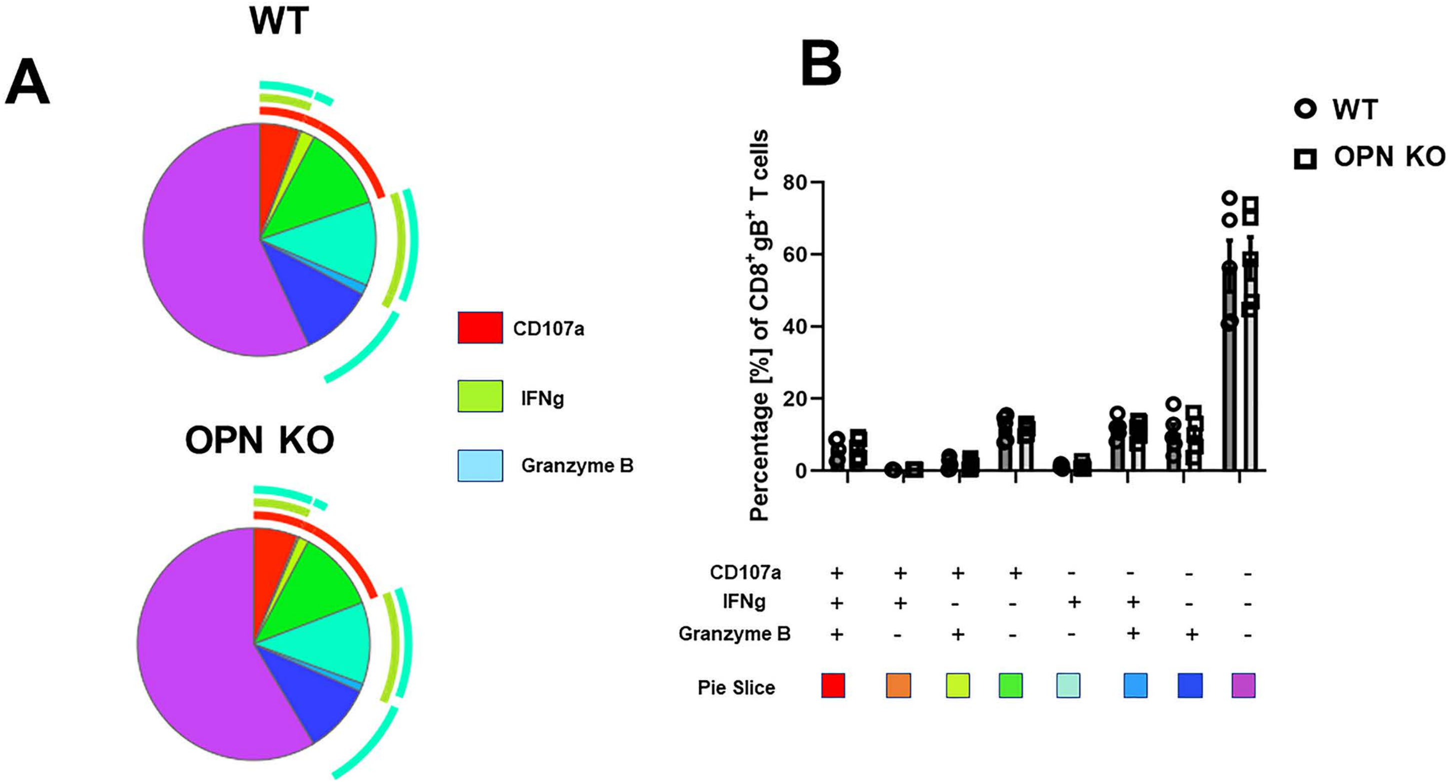
A
WT
OPN KO
CD107a
IFNg
Granzyme B
B
WT
OPN KO
Percentage [%] of CD8+gB+ T cells
80
60
40
20
0
CD107a + + + + - - - -
IFNg + + - - + + - -
Granzyme B + - + - - + + -
Pie Slice

Supplemental Figure 1. Wild type and OPN KO mice exhibit equivalent polyfunctional CD8$^+$ T cell response during acute ocular HSV-1 infection. Data generated from Figure 5 was used to assess for polyfunctionality of CD8$^+$ T cells using SPICE software with the results presented as pie charts in panel A. The average percentage of HSV-1 gB-specific CD8$^+$ T cells expressing CD107a, IFN-γ, and granzyme B from WT and OPN KO are displayed. The inner segment segments display cells expressing different combinations of proteins whereas the outer color-coded arcs around each circle show the frequency of cells expressing each protein (CD107a, IFN-γ, and granzyme B). The polyfunctionality of the cells is indicated in panel B with the data displayed as mean $\pm$ SEM.
